# Technical and Performance Characteristics Between Different Anti-Mullerian Hormone (AMH) Assay Methods and Antral Follicle Count (AFC) in Malaysian Women with Infertility: A University-Based Centre Cohort

**DOI:** 10.3390/life15030383

**Published:** 2025-02-28

**Authors:** Chong Jie Wen, Mohd Faizal Ahmad, Muhammad Azrai Abu, Nalisa Shamyra Johari, Izzatul Aliaa Badaruddin, Shah Shamsul Azhar, Abdul Kadir Abdul Karim

**Affiliations:** 1 Advanced Reproductive Centre (ARC), Hospital Canselor Tuanku Mukhriz (HCTM), Department of Obstetrics & Gynaecology, Faculty of Medicine, National University of Malaysia, Kuala Lumpur 56000, Malaysia; jiewenchong@hotmail.com (C.J.W.); drmohdfaizal@ukm.edu.my (M.F.A.); azraiabu1983@gmail.com (M.A.A.); myralissa@ukm.edu.my (N.S.J.); 2 Department of Pathology, Faculty of Medicine, National University of Malaysia, Kuala Lumpur 56000, Malaysia; izzatulaliaa@ukm.edu.my; 3 Department of Public Health, Faculty of Medicine, National University of Malaysia, Kuala Lumpur 56000, Malaysia; drsham@ppukm.ukm.edu.my

**Keywords:** anti-Mullerian hormone (AMH), antral follicle count (AFC), ovarian reserve

## Abstract

Backgrounds: Evaluating the AMH level and AFC are crucial in infertility practice. Thus, accurate measurement is paramount. Various methods are available and selecting the right process is essential to ensure good reproducibility and precise results. Thus, our study aims to determine the analytical performance of AMH Plus and AFIAS-AMH and the correlation between the two AMH assays and AFC values. Methods: A prospective study was conducted at the Advanced Reproductive Center, Hospital Canselor Tuanku Mukhriz (HCTM), Faculty of Medicine, National University of Malaysia, to compare the correlation between the AMH assay methods and AFCs. It included 100 patients from Feb 2024 until June 2024. Results: A total of 100 women with infertility, with a mean age of 35.87 ± 3.92, were included in our study. Our results revealed a strong positive correlation between the two AMH assays, with the comparable performance of AMH Plus and AFIAS-AMH. We also found that the serum AMH evaluation was comparable with the routine AFC assessment. Conclusions: Our findings indicated that serum AMH levels determine the association between AMH levels and follicle counts and the correlation between AMH concentrations and assays. Our study demonstrated the competent repeatability, acceptable linearity, and laboratory precision of the AFIAS-AMH test and comparative assessment of the AFC and serum AMH evaluation.

## 1. Introduction

A woman’s ovarian reserve, which is a crucial factor in determining her ability to procreate, is the quantity and quality of oocytes [1]. The assessment of ovarian reserves is increasingly crucial for managing and treating infertility. The findings enhance stimulation strategies and aid in counseling during fertility discussions and managing outcomes. An aging woman’s ovarian reserve is commonly known to deteriorate quantitatively and qualitatively, contributing to her decreased ability to procreate. Poor oogenesis and embryogenesis have been associated with mitochondrial dysfunction, often related to aging. However, in some cases, individuals may experience poor outcomes despite being younger, particularly in conditions like endometriosis with low ovarian reserve. As a result, assessing anti-Müllerian hormone (AMH) levels has become a standard practice. This evaluation helps anticipate the overall outcomes of stimulation and allows for tailored treatment approaches [2]. In clinical practice, anticipating an individual’s ovarian response before the initiation of an in vitro fertilization (IVF) cycle has important diagnostic and prognostic value [3]. Several methods for predicting ovarian reserve exist, such as the following: antral follicle count (AFC), follicle-stimulating hormone, and anti-Mullerian hormone (AMH) assays also known as Mullerian-inhibiting substance. The latter is secreted by the granulosa cells of preantral and small antral follicles and is used to measure ovarian reserves. It is crucial for forming follicles because it regulates autocrine and paracrine processes. Transforming growth factor beta contains AMH, a 140 kDa homodimer glycoprotein comprising two disulfide bonded, identical glycoprotein subunits [4,5]. The AMH value is highly reliable for predicting the ovarian response to ovarian stimulation and the prognosis for an IVF cycle because it is stable during intra- and intermenstrual cycles, has high sensitivity and specificity, and is a gonadotrophin-independent assessment of ovarian reserves [6,7,8]. To date, many AMH assays have been introduced, and either manual evaluation or automated assays have been implemented to measure serum AMH levels to provide good service and help in counseling, diagnosis, and treatment. Comparative studies have been conducted by using all the AMH assays and different methods in consideration of variations in criteria and regressions, enabling conclusions to be drawn on the basis of the correlations observed among these assays [9,10,11,12,13]. AFC is the number of ovarian follicles with diameters of less than 2–4 mm during the follicular phase. These follicles begin to develop after recruitment in the luteal phase and generally reflect the number of follicles that will potentially grow during the current menstrual cycle [14]. Most studies have shown that AMH is positively associated with AFCs [15]. To date, both the European Society of Human Reproduction and Embryology and the American Society for Reproductive Medicine agree that one ovarian reserve marker, either the antral follicle count (AFC) or anti-Müllerian hormone (AMH), should be assessed as a necessary evaluation before undergoing IVF treatment [16]. However, discordance between AMH levels and AFCs exists in clinical practice [17]. As a result, managing IVF cases will be challenging, mainly regarding regime formulation, expected responses, and overall prediction of IVF outcomes. Furthermore, misdiagnoses of high responders or poor responders also crucially interfere with overall IVF management in local practice [15]. Thus, it is vital to ensure this discrepancy is overcome. Changes in AFCs vary intra- and intercyclically, making them unfavorable for ovarian response prediction and correlations in clinical practice. Various methods are available for the evaluation of AMH levels, and their correlation with AFCs remains to be validated. Although antral follicle count (AFC) is considered the primary method for assessing ovarian reserve, we also include serum anti-Müllerian hormone (AMH) testing as part of our standard practice. Unfortunately, this testing is not offered in our university laboratory. As a result, we conduct AMH evaluations through standard serum assays at a private pathology laboratory; however, many women choose not to pursue this option due to cost concerns. Recently, we have been introduced to a more cost-effective method known as fluorescent immunoassay (FIA). Thus, our study aims to assess the accuracy and reproducibility of two different AMH assays, namely, AFIAS-AMH, which uses the fluorescent immunoassay (FIA) method (AFIAS POCT, BODITECH), and the standard AMH assay Elecys AMH Plus, which is based on electrochemiluminescence (ECLIA) (Cobas E, ROCHE). Our study aims to validate the correlation of AFIAS-AMH with the Elecys AMH Plus assay in terms of cost-effectiveness and overall AMH level results for the AFC evaluation of our patients for future clinical implementation.

## 2. Materials and Methods

### 2.1. Study Design

A prospective study was conducted at the Advanced Reproductive Center, Hospital Canselor Tuanku Mukhriz (HCTM), Faculty of Medicine, National University of Malaysia, to compare the correlation between the AMH assay methods and AFCs. All women who sought fertility assessment and fulfilled the criteria of being more than 18 years old without AMH in the past year from Feb 2024 until June 2024 were included regardless of the type of fertility, causes, and risk factors. The study was approved by the UKM Research Ethics Committee before the recruitment of subjects (UKM PPI/111/8/JEP-2023-992). All the patients enrolled in this study and their clinical profiles were recorded, with us using an Excel sheet as the data collection method. Subsequently, 3 mL of blood was collected from the women, divided into two samples, and evaluated by using two AMH assays: the standard test, namely, the Elecys AMH Plus assay based on ECLIA (Cobas E, ROCHE) at Hospital Pantai and the AFIAS-AMH based on FIA (AFIAS POCT, BODITECH) at the ARC HCTM Laboratory. To reduce operator bias, only one reproductive endocrinology fellow was tasked to perform a transvaginal ultrasound scan on menstrual days 1 to 4 to assess AFCs by using a single V7 ultrasound system machine with an EV2-10A transvaginal probe. In our setting, the early follicular phase assessment of AFC was chosen to detect the number of antral follicles 2–5 mm in diameter, which reflects the ovarian reserve and anticipated response to gonadotropin stimulation following the practical standardization [18].

### 2.2. Method Comparison

The Elecys AMH Plus assay based on ECLIA using the sandwich principle (Cobas E, ROCHE) was used. As a standard step, the first incubation was conducted with 50 µL of a sample, a biotinylated monoclonal AMH-specific antibody, and a monoclonal AMH-specific antibody labeled with a ruthenium complex to form a sandwich complex. After streptavidin coated microparticles were added during the second incubation, the molecules activated the interaction between biotin and streptavidin. An electrode was used to capture the reading. Next, the voltage of the electrode induced chemiluminescent emission, which was measured by a photomultiplier. The results were determined by using an instrument generated calibration curve that was generated explicitly by two-point calibration and a master curve provided by a reagent barcode or e-barcode. The total duration of the assay was 18 min. The measurement range was 0.14–107 pmol/L (0.02–15 ng/mL). According to the EP 09-A3 protocol of the Clinical Laboratory Standards Institute, each group for a method comparison study requires at least 40 samples. A large number of samples will improve the results and reduce the unnecessary bias of the final result interpretation. Therefore, in our study, 100 women were recruited. All collected data were analyzed by using Python software 3.10.12. For method comparison, the data acquired were analyzed by using either a difference or comparison plot on the basis of their agreement. Subsequently, the data were analyzed for the association between the assays and AFCs by using regression analysis. The within run and total imprecisions of the Boditech AFIAS POCT were expressed as the mean and coefficient variation to assess outcomes. Linear regression analysis was conducted to compare the Roche Cobas E analyzer and FIA-based assay (AFIAS POCT, BODITECH). Slope, intercept, correlation coefficient (r), coefficient of determination (r^2^), and bias were determined. The correlation between the AMH levels and AFCs was determined by using Spearman’s rank coefficient. For quality control (QC), procedures were performed over three days, with QC performed two times daily for level 1 (QC range 0.69–0.86) and level 2 AMH (QC range 6.39–7.99) on the basis of the established data for the QC FIA (AFIAS POCT, BODITECH) analyzer in AMH assessment.

## 3. Results

**Figure 1:**
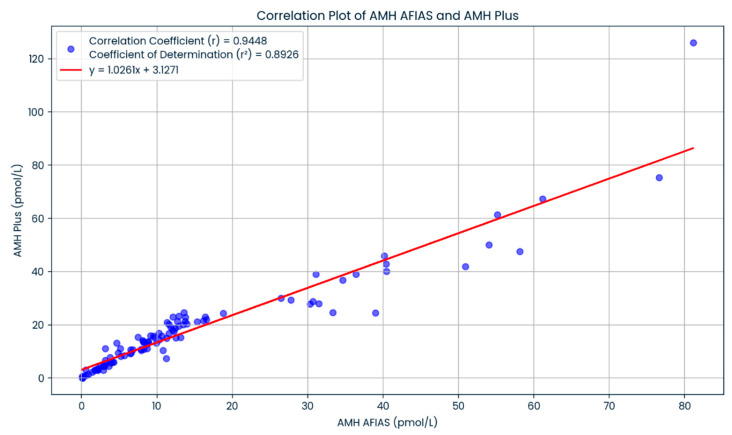
The correlation of the results of AFIAS-AMH and AMH Plus.

**Figure 2:**
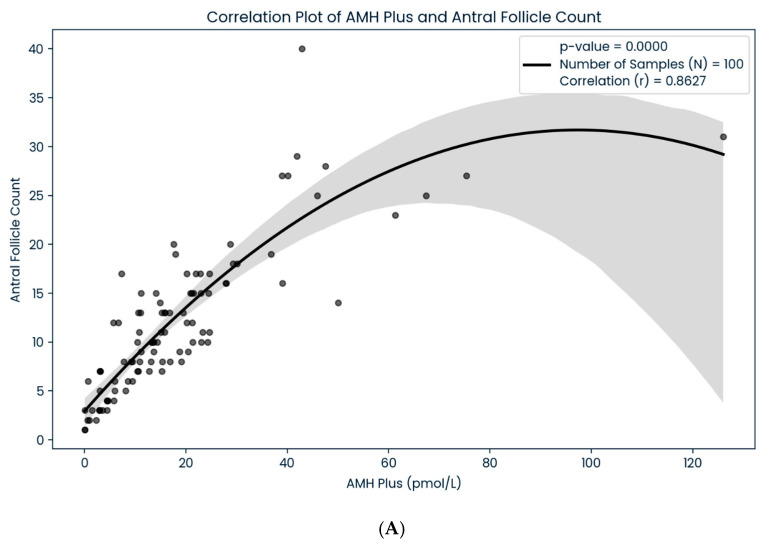

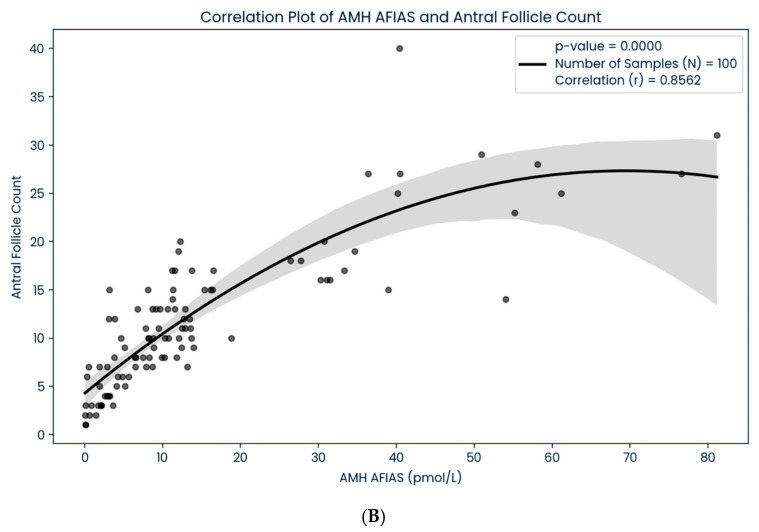
(**A**): The correlation between antral follicle count and AMH Plus. (**B**): The correlation between antral follicle count and AFIAS-AMH.

**Figure 3:**
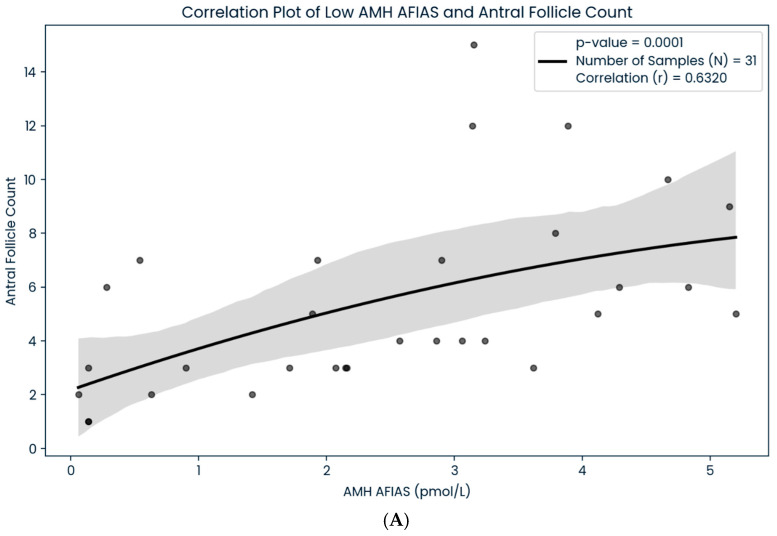

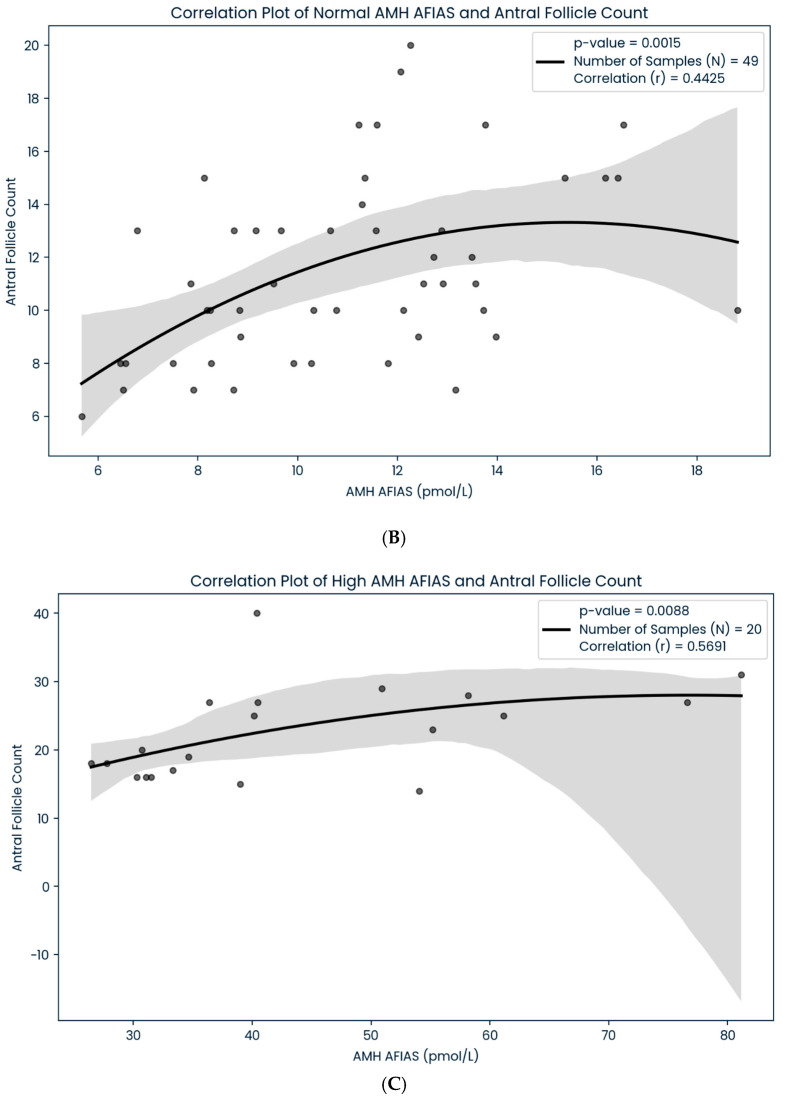
(**A**): The correlation between AFIAS-AMH in low ovarian reserve study population. (**B**): the correlation between AFIAS-AMH in normal ovarian reserve study population. (**C**): the correlation between AFIAS-AMH in high ovarian reserve study population.

A total of 100 women with infertility, with a mean age of 35.87 ± 3.92, were included in our study. The majority of the patients in our analysis were Malay (81%), followed by Chinese (12%), Indians (6%), and other ethnicities (1%), in that order. Women with obesity (BMI > 27.5 kg/m^2^), overweight (BMI 23–27.5 kg/m^2^), average weight (BMI 18.5–23 kg/m^2^), and underweight (BMI < 18.5 kg/m^2^) accounted for 40%, 33%, 26%, and 1% of the total sample, respectively (Table 1). In addition to that, we found a significant correlation between the AMH Plus and AFIAS-AMH assay technique in subgroups of women depending on their ovarian reserve category (*p* < 0.05) (Table 2). Our data showed that the regression line had a slope of 1.0261 and an intercept of 3.1271 (r^2^ = 0.8926), which revealed a strong positive correlation between the two AMH assays (Figure 1). The results suggested that both methods are consistent and comparable in terms of analytical performance. Moreover, we also found that both the AFIAS-AMH and AMH Plus showed a significant correlation regarding AFCs (*p* < 0.05) (Figure 2). Nevertheless, the AFIAS-AMH showed significant positive correlations with AMH Plus in the sub analysis of women with low (Figure 3A), normal (Figure 3B), and high ovarian reserves (Figure 3C).

## 4. Discussion

As established, assessing serum AMH levels has become vital before IVF treatment in predicting ovarian response. Most evidence indicates that AMH levels are a good and reliable measure of ovarian reserve. Nevertheless, most centers adopt various methods for AMH assay evaluation, leading to a lack of standardization mainly due to the cost issues. In government IVF centers, patients often pay for AMH evaluation, leading to more AFC evaluations than AMH. Thus, not surprisingly, various AMH assay methods have been proposed to address this problem. However, these have aroused concerns about the consistency and reliability of their results, leading to challenges in interpreting test results and data comparisons across studies [19]. Similarly, in our center, we adopted an alternative method and compared it to the standard method to ensure that the consistency and reliability of both results were comparable before clinical implementation. Our study revealed a similar solid linear correlation between the AMH values of two assay methods (Elecys and AFIAS) [1,3]. The results showed high correlation and consistency between these two assays, most likely due to the same biological variable. In comparison, some studies have stated a precise percentage of discrepancy between laboratories, and no standard international criteria for new automatized assays exist. Therefore, concerns about potential biases and result deviations have arisen [4,20]. A previous study compared two AMH assays, namely, Access2 and DxI800 analyzers, to evaluate sample stability and intra- and interassay interpretation. They found that the differences between the two methods were within clinically acceptable ranges, with a strong correlation which suggested that both methods are interchangeable [10].

As elaborated, in the center, access to serum AMH evaluation is restricted, primarily due to financial constraints; the centralization of laboratories is often adopted, resulting in lengthy waiting times for results and suboptimal clinical practice similar to our local practice. Our center opted for the alternative, rapid, interpretative, laboratory AFIAS-based assay utilizing serum AMH with a short turnaround time to overcome this. We propose the alternative method as a good strategy for solving the above issues. In our center, the AFIAS-AMH is conducted by using the available automated POCT AMH assay, which is reasonably priced, to obtain on-site laboratory results in a simple setting within the reproductive center itself [21,22]. In terms of the consistency of the results, our study showed that AFIAS-AMH maintained a substantial positive correlation even after the sub analyses of three categories of ovarian reserves: low (<5.4 pmol/L), average (5.5–24.9 pmol/L), and high (>25 pmol/L). Furthermore, AFIAS-AMH exhibited a good correlation with AMH Plus. The optimal AMH level is often used as a guide for clinicians to formulate the medication dosage for women undergoing IVF, aiming for personalized care and individualized decisions. On the other hand, the AMH levels have been reported to be substantially linked with AFCs [9]. At least 90% of the population, translating to approximately one in five women with infertility, show discordant AFCs and AMH levels [23]. Therefore, AFCs are preferred for predicting ovarian response due to this disparity in results and the final oocyte yield [15,24,25]. Nevertheless, the selection of women for AMH and AFC evaluation is paramount, with the bias control of operators for AFC evaluation being vital to ensure a reduction in discrepancies between AMH and AFC evaluations. To date, the evidence has been reported that AFCs and AMH show a negative correlation within the advanced women cohort in regard to the correlation of AFC evaluation and serum AMH [26]. In depth, various studies have reported that AFCs decline linearly with age, whereas others have reported that AFCs decline biphasically [27,28,29]. The evidence observed a significant variation with age throughout the menopausal phase that may be attributed to differences in ovarian reserves at various ages. Despite age, the discordance between AMH levels and AFCs in infertile women can be attributed to multiple factors, such as body mass index, socioeconomic status, and environmental/nutritional factors, like vitamin D status. Nevertheless, in women with PCOS, the range of serum AMH levels do affect the interassay correlation and association between AMH levels and follicle counts. Overall, the correlation between AFCs and AMH levels remains inconclusive. The technical limitations in follicle counting, such as the bias of assessment and analytical variability of the AMH assay used, also contribute to this discrepancy between the AFC and level of AMH. The technical limitations in follicle counting, such as the bias of assessment and analytical variability of the AMH assay used, also contribute to this discrepancy between the AFC and the level of AMH. Therefore, additional robust data should be made available to evaluate this correlation. Again, this result underlines the importance of standardizing and accurately interpreting AMH tests in clinical settings [21]. As a practice, one ovarian reserve marker, either the AFC or AMH, can be opted for evaluation before undergoing IVF treatment based on the ESHRE or ASRM recommendation which is based on the local setting and preferences. In our study, we examined the correlation between sonographic (AFC) and endocrine (AMH) markers to determine reproductive potential. Our findings support the fact that when the correlation between AFCs and AMH levels was evaluated using two distinct assays it was found to be comparable. We found that the linear correlation between AFCs and AMH levels determined by two different assays is interchangeable. Thus, our findings suggested that the number of antral follicles influences the serum AMH level. Therefore, per local routine practice, we opted for serum AMH as an essential clinical parameter to predict the overall IVF cycle outcome. Using the current method of AFI-AS-based assay, our evaluation is cost-effective and timely, reducing the operator-dependent bias via AFC assessment.

Nevertheless, as our limitation, our sample size is considered small and reflects a single-center experience. Therefore, multi-center trials with bigger sample sizes should be proposed to ensure reproducibility and consolidation of the overall outcome. In addition, exploring various methods of serum AMH assays could be a way forward compared to standard serum AMH assay assessment to ensure variability for future options. To date, technical research that aims for better quality and cost in serum AMH evaluation can be proposed as an outcome that can be adopted for future clinical implementation.

## 5. Conclusions

Our findings indicated that serum AMH levels determine the association between AMH levels and AFC and the correlation between the two types of AMH evaluation. Our study demonstrated the competent repeatability, acceptable linearity, and within-laboratory precision of the AFIAS-AMH test compared to the standard AMH assessment, regardless of the women’s ovarian reserve category. Consequently, we recommend that the AFIAS-AMH assay be a substitute as an alternative immunoassay in our local center to ensure cost-effectiveness and timely effectiveness for better clinical implementation in our IVF practice.

## Figures and Tables

**Table 1:** Description of demographics of study population.

Parameter	Percentage (%)
Age, years old	
<35	38 (38%)
≥35	62 (62%)
Race	
Malay	81 (81%)
Chinese	12 (12%)
Indian	6 (6%)
Lain—lain	1(1%)
BMI, kg/m^2^	
Underweight (<18.5)	1 (1%)
Normal (18.5–23.0)	26 (26%)
Overweight (23.0–27.5)	33 (33%)
Obesity (>27.5)	40 (40%)
Type of Infertility	
Primary	77 (77%)
Secondary	23 (23%)
Cause of Infertility	
PCOS	17 (17%)
Endometriosis	24 (24%)
Uterine fibroid	1 (1%)
Tubal	13 (13%)
Male	19 (19%)
Unexplained	26 (26%)

**Table 2:** Summary of the correlation for the subgroup (low, normal, and high ovarian reserve).

Scheme 100	AFIAS-AMH (*n*:100) Correlation (r)	AMH Plus (*n*:100) Correlation (r)	*p* Value
Low (<5.4 pmol/L)	0.6320 (n: 31)	0.4516 (n:20)	*p* < 0.05
Normal (5.5–24.9 pmol/L)	0.4425 (n: 49)	0.5135 (n:62)	*p* < 0.05
High (>25.0 pmol/L)	0.5691 (n:20)	0.5965 (n:18)	*p* < 0.05

## Data Availability

The original contributions presented in this study are included in the article. Further inquiries can be directed to the corresponding author.
